# Frequency, characteristics, and immunological accompaniments of ataxia in anti-NMDAR antibody-associated encephalitis

**DOI:** 10.3389/fimmu.2024.1500904

**Published:** 2024-12-13

**Authors:** Sarah Jesse, Marie Riemann, Hauke Schneider, Marius Ringelstein, Nico Melzer, Niklas Vogel, Lena Kristina Pfeffer, Manuel A. Friese, Kurt-Wolfram Sühs, Dominica Hudasch, Philipp Schwenkenbecher, Albrecht Günther, Christian Geis, Jonathan Wickel, Martin Lesser, Annika Kather, Frank Leypoldt, Justina Dargvainiene, Robert Markewitz, Klaus-Peter Wandinger, Franziska S. Thaler, Joseph Kuchling, Katharina Wurdack, Lidia Sabater, Carsten Finke, Jan Lewerenz

**Affiliations:** ^1^ Department of Neurology, University Hospital Ulm, Ulm, Germany; ^2^ Department of Neurology, Augsburg University, Augsburg, Germany; ^3^ Department of Neurology, Medical Faculty, Heinrich Heine University of Düsseldorf, Düsseldorf, Germany; ^4^ Department of Neurology, Centre for Neurology and Neuropsychiatry, LVR-Klinikum, Heinrich-Heine-University Düsseldorf, Düsseldorf, Germany; ^5^ Institute of Neuroimmunology and Multiple Sclerosis and Department of Neurology, University Medical Center Hamburg-Eppendorf, Hamburg, Germany; ^6^ Department of Neurology, Hannover Medical School, Hannover, Germany; ^7^ Section of Translational Neuroimmunology, Department of Neurology, Jena University Hospital, Jena, Germany; ^8^ Department of Neurology, Carl Gustav Carus University Dresden, Dresden, Germany; ^9^ Institute of Clinical Chemistry, University Hospital Schleswig-Holstein, Kiel/Lubeck, Germany; ^10^ Department of Neurology, University Hospital Schleswig-Holstein, Kiel, Germany; ^11^ Institute of Clinical Neuroimmunology, LMU University Hospital, LMU Munich, Munich, Germany; ^12^ Biomedical Center (BMC), Faculty of Medicine, LMU Munich, Munich, Germany; ^13^ Department of Neurology and Experimental Neurology, Charité – Universitätsmedizin Berlin, Berlin, Germany; ^14^ Fundació de Recerca Biomèdica Clínic Barcelona-Institut d’Investigacions August Pi i Sunyer-Caixa Research Institute, Universitat de Barcelona, Barcelona, Spain; ^15^ Spanish National Network for Research on Rare Diseases (CIBERER), Madrid, Spain

**Keywords:** NMDAR-encephalitis, ataxia, outcome, cerebellum, multiple sclerosis, MOG antibody, aquaporin-4 antibody

## Abstract

**Introduction:**

Very rarely, adult NMDAR antibody-associated encephalitis (NMDAR-E) leads to persistent cerebellar atrophy and ataxia. Transient cerebellar ataxia is common in pediatric NMDAR-E. Immune-mediated cerebellar ataxia may be associated with myelin oligodendrocyte glycoprotein (MOG), aquaporin-4 (AQP-4), kelch-like family member 11 (KLHL11), and glutamate kainate receptor subunit 2 (GluK2) antibodies, all of which may co-occur in NMDAR-E. Here, we aimed to investigate the frequency, long-term outcome, and immunological concomitants of ataxia in NMDAR-E.

**Methods:**

In this observational study, patients with definite NMDAR-E with a follow-up of >12 months were recruited from the GENERATE registry. Cases with documented ataxia were analyzed in detail.

**Results:**

In 12 of 62 patients (19%), ataxia was documented. Bilateral cerebellar ataxia without additional focal CNS findings was found in four (one child and three adults); one of these was previously reported as a case with persistent cerebellar atrophy and ataxia. Two patients with bilateral cerebellar ataxia had additional focal neurological symptoms, optic neuritis and facial palsy. Two patients developed hemiataxia: one with diplopia suggesting brainstem dysfunction and the other probably resulting from cerebellar diaschisis due to contralateral status epilepticus. In all but the one developing cerebellar atrophy, cerebellar ataxia was transient and not associated with a worse long-term outcome. In all five patients with cerebellar ataxia tested, MOG, AQP-4, GluK2, and KLHL11 antibodies were negative. In two additional patients negative for both MOG and AQP-4 antibodies, ataxia was sensory and explained by cervical myelitis as part of multiple sclerosis (MS) manifesting temporal relation to NMDAR-E. One of the patients with bilateral ataxia with focal neurological deficits was also diagnosed with MS upon follow-up. Finally, in two patients, ataxia was explained by cerebral hypoxic damage following circulatory failure during an ICU stay with severe NMDAR-E.

**Discussion:**

Ataxia of different types is quite common in NMDAR-E. Cerebellar ataxia in NMDAR-E is mostly transient. NMDAR-E followed by persistent ataxia and cerebellar atrophy is very rare. Cerebellar ataxia in NMDAR-E may not be explained by concomitant KLHL11, MOG, AQP-4, or GluK2 autoimmunity. Of note, ataxia in NMDAR-E may result from treatment complications and, most interestingly, from MS manifesting in temporal association with NMDAR-E.

## 1 Introduction

N-methyl D-aspartate receptor (NMDAR) antibody-associated encephalitis (NMDAR-E) is an autoimmune-encephalitis subtype characterized by the intrathecal synthesis of IgG antibodies against the NMDAR subunit NR1 (1). Anti-NMDAR IgG in NMDAR-E results in immunoglobulin-induced NMDAR internalization and thus inhibition of NMDAR signaling (2). Consequently, some symptoms as well as the changes in cerebral glucose metabolism upon positron-emission tomography resemble pharmacological NMDAR inhibition (3). Unless early immunosuppression is initiated, the clinical course of NMDAR-E follows a characteristic pattern: after a flu-like prodromal phase, psychotic symptoms develop, followed by speech disturbances and epileptic seizures, then—interpreted as generalized encephalitis—loss of consciousness, stereotypic movements, and autonomic dysfunction as well as central hypoventilation (4). The clinical picture of NMDAR-E varies with age: in infants and young children, movement disorders might predominate, and autonomic dysfunction and hypoventilation are less common (5). In addition, cerebellar ataxia and focal neurological deficits have been reported to be much more common in children than in adults with NMDAR-E (6). However, NMDAR-E in adulthood may present initially as cerebellar ataxia (7).

Of note, NMDAR subunit NR1 is expressed in the human (8) and rodent cerebellum (9). NMDAR antagonists are known to induce impaired motor coordination in mice (10) and non-human primates (11). In rats, prominent changes of cerebellar NMDAR subunit expression from birth to adulthood were demonstrated (9). Whether similar changes occur during childhood in humans is unknown. However, this could explain the high frequency of ataxia reported for childhood NMDAR-E.

We recently reported a case with severe NMDAR-E, which resulted in persistent severe cerebellar ataxia associated with progressive cerebellar atrophy (12). A few similar cases had been reported previously by Iizuka et al. (13). This rare phenotype of unknown pathophysiology, herewith coined a Iizuka phenotype, is characterized by cerebellar atrophy associated with post-acute reversal of supratentorial atrophy (12, 13). Severe persistent atrophy explains poor functional long-term outcome in these patients (12, 13).

NMDAR-E, including cases with focal neurological deficits suggestive of demyelinating events, has been associated with MOG and AQP-4 antibodies in some patients (14, 15). Neuromyelitis optica spectrum disorders (NMOSD) with AQP-4 antibodies as well as MOG-antibody associated diseases (MOGAD) may present with ataxia (16). Recently, two antibodies, directed against kelch-like family member 11 (KLHL11) and glutamate kainate receptor subunit 2 (GluK2), also associated with cerebellar ataxia, have been described (17, 18). Interestingly, KLHL11 antibodies share their association with ovarian teratomas with NMDAR antibodies (17, 18).

Here, we investigated the frequency of ataxia in mostly adult NMDAR-E patients enrolled in the multicentric registry of the German Network for Research on Autoimmune Encephalitis (GENERATE). In addition, we analyzed the detailed clinical characteristics, the immunological accompaniments, and the long-term functional outcome of ataxia associated with NMDAR-E.

## 2 Methods

### Study design and participants

GENERATE is a multicentric, combined retrospective and prospective registry for autoimmune encephalitis patients in Germany (https://generate-net.de/) recruiting since 2013. The inclusion criteria for this analysis were as follows: (a) diagnosis of definite NMDAR-E according to current diagnostic criteria with detection of anti-NMDAR IgG (19) with the modification that patients with consistently negative anti-NMDAR IgG in cerebrospinal fluid were excluded; (b) available information on initial and predominating clinical symptoms; and (c) available long-term functional outcome (≥12 months) as a modified Rankin scale (mRS) score. A study plan was sent to the GENERATE sites in April 2017. Thirty-one sites agreed to participate. The recruitment period ended in February 2018. Clinical information was extracted from the database of the GENERATE registry. For patients with documented ataxia, more detailed information was collected in close collaboration with the respective GENERATE sites. This detailed information is summarized as case vignettes to be found in the online appendix. MRIs of all ataxia patients were centrally re-analyzed by experts in NMDAR-E and MS imaging (J.K., K.W., and C.F.) to detect ataxia-associated abnormalities as well as to confirm the diagnostic criteria for MS (20).

### Antibody testing

Sera of atactic patients with serum samples still available in the GENERATE biomaterial banks were tested using in-house live cell–based assays for MOG and GluK2, in-house paraformaldehyde-fixed in-house cell-based assay for KLHL11 antibodies, all three with transiently transfected HEK293 cells, and a commercial HEK293-based assay from Euroimmun (Luebeck, Germany) for AQP4 antibodies as described previously (17, 18, 21, 22). NMDAR antibodies and all antineuronal antibodies obtained during the initial diagnostic workup of the patients with ataxia and NMDARE were obtained from the clinical files of the patients. Serum of Patient 10 had been tested by commercial cell-based assays from Euroimmun in serum only, but never in CSF. Thus, we confirmed anti-NMDAR IgG using paraformaldehyde-fixed rat brain sections as described previously (23).

### Statistics

Statistical analysis was performed using GraphPad Prism (Graph Pad Inc., La Jolla, USA). Categorical variables were analyzed using Fisher’s exact test. For ordinal and continuous variables, the median and the interquartile range (IQR) were calculated, and the Mann-Whitney U test was used to compare two groups. A two-sided *p*-value of <0.05 was regarded as statistically significant. Due to the explorative nature of this study, all results from statistical tests must be interpreted as hypothesis generating.

### Data availability

The datasets generated and/or analyzed during the current study are not publicly available but can be obtained by qualified researchers from the corresponding author upon reasonable request.

### Ethics

All patients or their legal representatives gave their informed consent. The study was approved by the institutional review board of the University of Schleswig-Holstein (#13-162).

## 3 Results

### Study cohort

**Figure 1:**
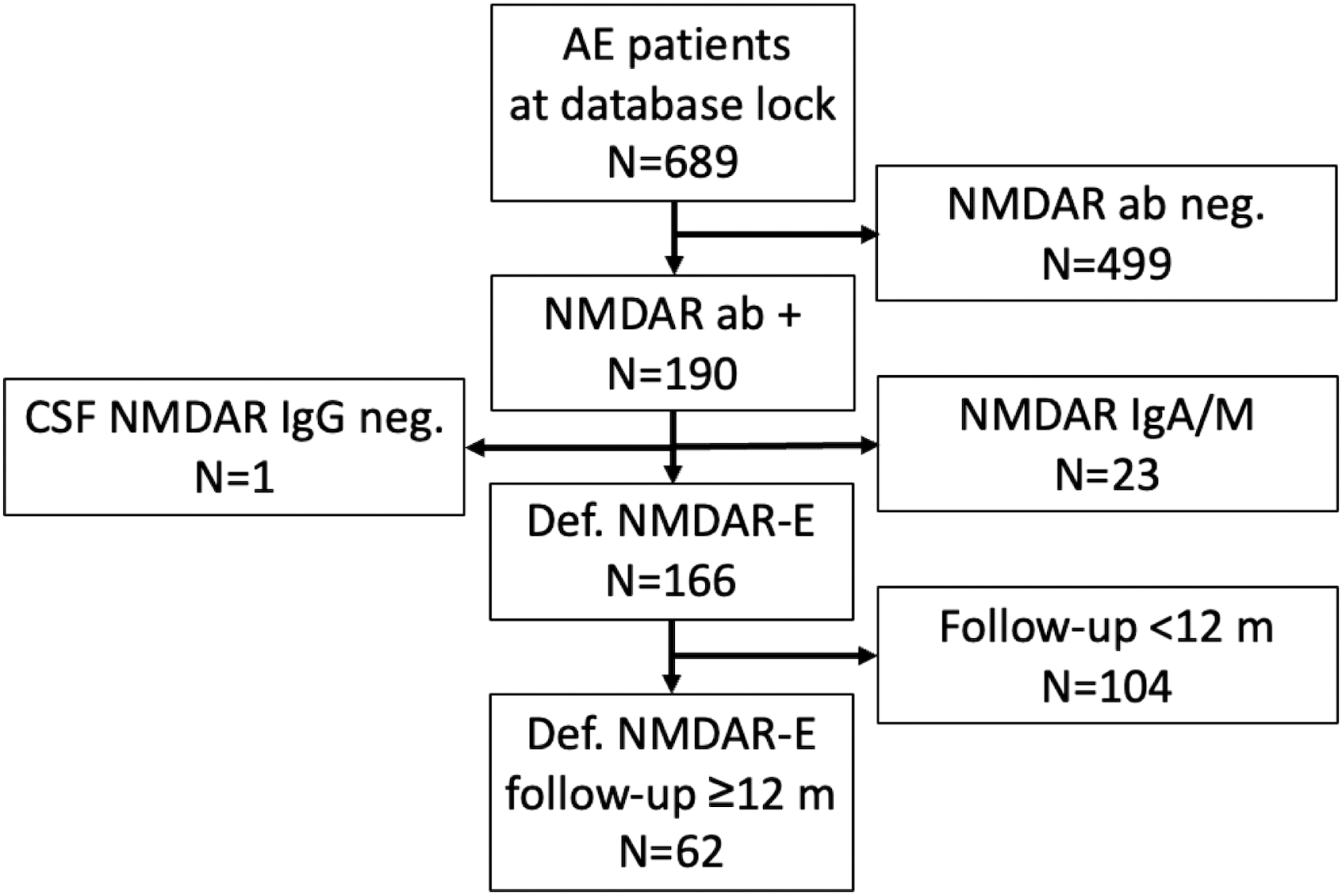
Study recruitment. AE, autoimmune encephalitis; ab, antibody; CSF, cerebrospinal fluid; NMDAR-E, N-methyl D-aspartate receptor antibody-associated encephalitis.

At database cut, 689 patients had been enrolled in the GENERATE registry (**Figure 1**). Of those, 190 (28%) had been documented positive for NMDAR antibodies. Twenty-four patients were excluded as they did not meet the diagnostic criteria of definite NMDAR-E as described in the inclusion criteria. Anti-NMDAR IgG in CSF was repeatedly negative, although serum anti-NMDAR IgG was positive in one patient (4%). Other reasons for exclusion were anti-NMDAR IgA or IgM only (*n* = 23, 12%). Overall, definite NMDAR-E was confirmed in 166 cases (85%). In 62 of these patients (37%), follow-up data for functional impairment as mRS score were available for at least 12 months after initial hospital admission. In our patient collective, six patients (10%) in the total cohort were younger than 18 years; only one (2%) was younger than 12 years. The basic demographic variables of this cohort can be found in **Table 1**.

**Table 1:** Demographic data of total patient cohort.

	Total cohort (*n* = 62)
Demographic data	
**Age (years),** median (IQR)	25 (21–40)
**Female:male,** *n* (%)	49:13 (79:21)
Disease severity	
**Acute phase**	
**Duration, days,** median (IQR)	49 (26–85)
**Max. mRS,** median (IQR)	4 (3–5)
**mRS ≥ 3,** *n* (%)	56 (90)
**Mechanical ventilation,** n (%)	15 (24)
Dismissal from hospital	
**mRS,** median (IQR)	2 (2–3)
**mRS ≥ 3,** *n* (%)	26 (42)
Last follow-up ≥ 12 months after disease onset	
**months,** median (IQR)	14 (12–17)
**mRS,** median (IQR)	1 (0–2)
**mRS ≥ 3,** *n* (%)	9 (15)
CSF analysis	
**WBC/µl,** median (IQR)	16 (4–44)
**Pleocytosis,** *n* (%)	46 (74)
**Positive oligoclonal bands,** *n* (%)	43 (69)
Further findings	
**Tumor,** *n* (%)	10 (16)
**Pathologic MRI,** *n* (%)	37 (60)

*n*, number of patients; IQR, inter-quartile range; mRS, modified Rankin Scale; CSF, cerebro-spinal fluid; WBC, white blood cells; MRI, magnetic resonance imaging.

### Different types of ataxia due to diverse pathophysiologies occur with different temporal association to NMDAR-E

In addition to our index case with the progressive cerebellar atrophy associated with severe and persistent cerebellar ataxia, following severe NMDAR-E (Case 4) (12), a phenotype that due its first describer (13) we suggest to name Iizuka-type NMDAR-E, another 11 patients were documented as exhibiting ataxia during the acute NMDAR-E episode in the GENERATE database. None of these tested positive for other antibodies than anti-NMDAR during routine work-up (**Supplementary Table S1**).

**Figure 2:**
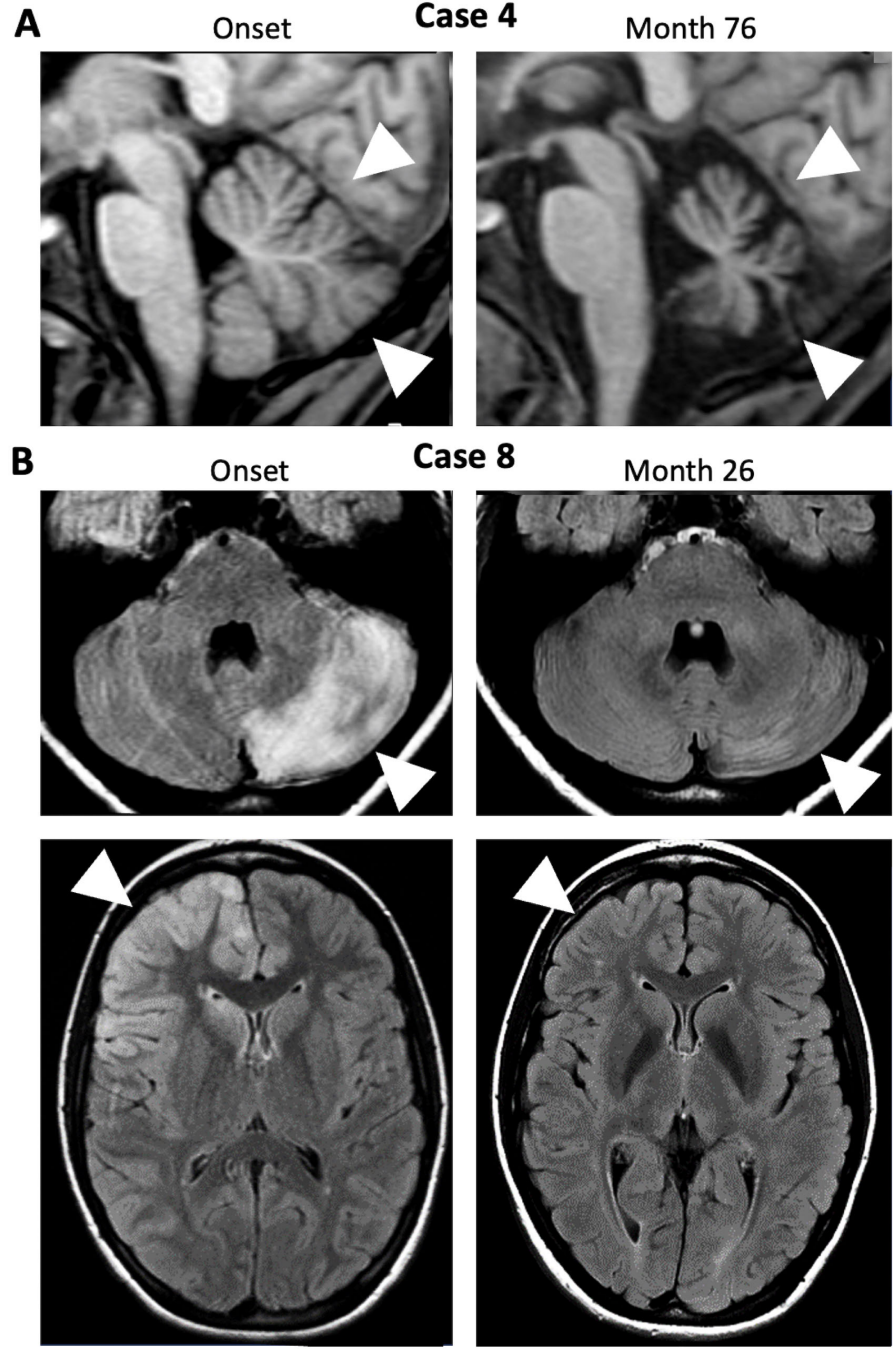
MRI changes in NMDAR-E patients with cerebellar ataxia are rare. **(A)** Sagittal 1-weighted MRI of Case 4 at onset (left panel) and after recovery from severe NMDAR- E but with persisting bilateral cerebellar ataxia (month 76; arrows indicate cerebellar atrophy). **(B)** FLAIR images of Case 8 with reversible left-sided hemiataxia in the acute phase (onset, left panels) show diffuse, mostly cortical left cerebellar T2-hyperintensity (upper left panel, arrowhead) possibly resulting from cerebellar diaschisis following right frontal status epilepticus associated with right frontal cortical hyperintensities (lower panel, arrowhead). T2-hyperintensities were reversible (Month 26, right panels.

A detailed analysis of these cases, including the index case, allowed categorization into five different groups (**Table 2**). The first group consisted of four patients who presented with bilateral cerebellar ataxia in close temporal relationship to the onset of symptoms typical for the NMDAR-E episode without additional focal CNS symptoms beyond ataxia. In the youngest patient of our cohort, a 7-year-old child, bilateral symmetric cerebellar ataxia was the first symptom of NMDAR-E (Case 1). Two other adult patients developed bilateral cerebellar ataxia after the onset of typical NMDAR-E symptoms (Cases 2–3). All of these patients had a good prognosis, including complete remission of the ataxia. In contrast, the patient with Iizuka-type NMDAR-E upon long-term follow-up never reached an mRS<4 due to severe persistent cerebellar ataxia associated within progressive cerebellar atrophy (**Figure 2A**) after she had recovered from all other symptoms directly related to the NMDAR-E episode. Initially, prolonged analgesia was required with numerous complications during intensive care (12).

**Table 2:** Groups of NMDAR-E patients with documented ataxia.

Case	Age (years)	Sex	Timing vs. typical NMDAR-E	Additional clinical and laboratory characteristics	Outcome at 12 months		Imaging findings	MOG/ AQP-4/ KLHL11/GluK2 Abs
					NMDAR-E	Ataxia		
Bilateral cerebellar ataxia without focal CNS abnormalities								
1	7	f	prior	childhood NMDAR-E	good	good	normal	n.d.
2	46	f	delayed	NMDAR Abs initially neg., relapse of ataxia, high CSF lactate	good	good	normal	n.d.
3	29	f	delayed	none	good	good	normal	-/-/-/-
4	23	f	delayed	sepsis, PRES, CIM, probable mitochon-drial toxicity	good	poor	progressive cerebellar atrophy	-/-/-/-
Bilateral cerebellar ataxia with focal CNS abnormalities with/without multiple sclerosis								
5	39	f	prior	preceding visual disturbance left eye, relapses of ataxia	good	good	normal	-/-/-/-
6	21	f	prior	clumsiness left hand, palatal numbness, right facial palsy	good	good	pericallosal T2w hyperintensity	-/-/n.d.
Cerebellar hemiataxia								
7	21	f	early	diplopia	good	good	left cortical/ temporomesial DWI lesion	-/-/-/-
8	18	f	delayed	NMDAR abs initially negative, MRZ+, Cerebellar diaschisis	good	good	diaschisis: right frontal/left cerebellar hyperintensity	-/-/-/-
Sensory ataxia associated with multiple sclerosis								
9	59	m	prior	NMDAR abs initially neg., MRZ+	good	good	white matter lesions cerebral and myelon	-/-/n.d.
10	40	f	prior	diplopia, persistent spastic tetraparesis	good	poor	white matter lesions cerebral and myelon	-/-/n.d.
Symptomatic ataxia associated with cerebral hypoxic-ischemia								
11	20	f	delayed	sepsis, resuscitation, acute respiratory distress syndrome	good	good	bilateral cerebellar hypoxic-ischemia	n.d.
12	33	m	delayed	resuscitation, prolonged weaning	good	good	bilateral ischemia occipital/central	-/-/n.d.

NMDAR-E, N-methyl D-aspartate receptor antibody-associated encephalitis; MOG, AQP-4, aquaporin-4; KLHL11, Kelch-like family member 11; GluK2, glutamate kainate receptor subunit 2; CNS, central nervous system; MRZ, measles, rubella, zoster.

The second group consisted of two patients, both of whom developed bilateral cerebellar ataxia in addition to other focal neurological deficits beyond typical NMDAR-E symptoms. One patient reported a monocular decrease in visual acuity many months before the onset of ataxia and then NMDAR-E-typical symptoms (Case 5). The other patient, a 21-year-old woman, developed mild facial paralysis, palatal hypoesthesia, and clumsiness of the left hand in close temporal association with cerebellar ataxia and NMDAR-E-typical symptoms (Case 6).

In the third group, patients also showed other focal neurological abnormalities, but cerebellar hemiataxia instead of bilaterally symmetric ataxia. In the first patient (Case 7), a 21-year-old woman, cerebellar hemiataxia developed in parallel with diplopia and NMDAR-E-typical symptoms. Although the combination of diplopia and hemiataxia suggests a brainstem lesion, cerebral MRI was unremarkable. In the other patient (Case 8), an 18-year-old woman, hemiataxia developed later in the disease course. Interestingly, in this patient, cerebral MRI showed a prominent T2-weighted lesion of the cerebellum involving almost the entire hemisphere ipsilateral to the ataxia (**Figure 2B**). This lesion was located contralateral to the right frontal T2-weighted hyperintensities, predominantly cortical, but also extending into the underlying white matter as an MRI correlate of focal status epilepticus. All lesions were transient. Thus, the hemiataxia can be interpreted as symptomatic cerebellar diaschisis secondary to status epilepticus (24) directly caused by NMDAR-E.

**Figure 3:**
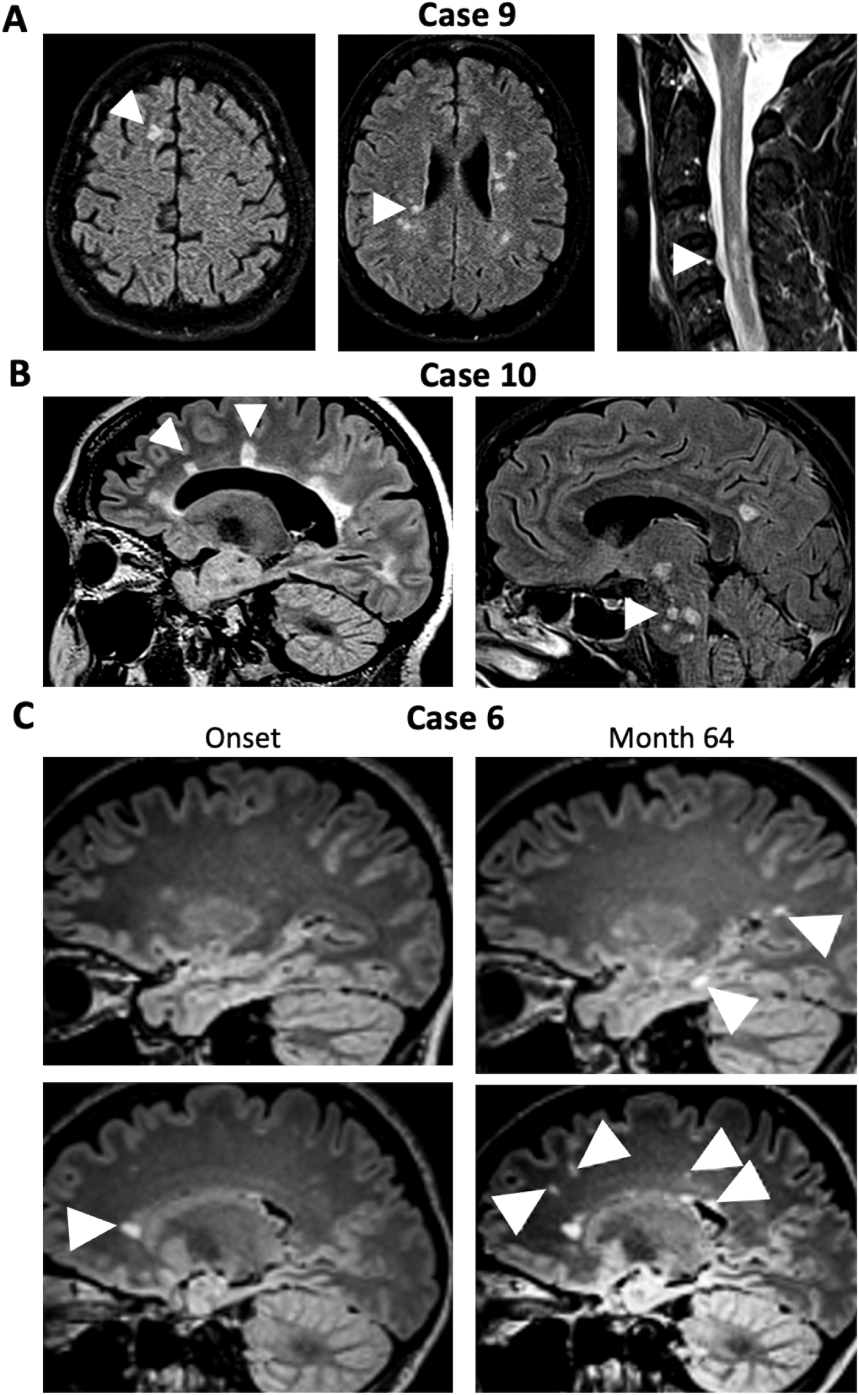
MRI in patients NMDAR-E with ataxia and concomitant or later diagnosis of multiple sclerosis. **(A)** Initial FLAIR cerebral MRI of Case 9 show juxtacortical (left panel, arrowhead) as well as periventricular lesions (middle panel, arrowhead) suggestive of demyelination. T2-weighted MRI of the cervical spinal cord also shows multiple demyelinating lesion (arrowhead). **(B)** Cerebral FLAIR MRI of Case 10 shows extensive periventricular T2-hyperintense lesions with Dawson finger-like orientation, highly suggestive of MS-like demyelination (left panel, arrowhead) as well as infratentorial lesions (right, arrowhead). **(C)** Initial cerebral FLAIR MRI of Case 6 (left panels) shows small unspecific subcortical white matter lesions but one periventricular lesion (arrowhead). Upon follow-up (Month 64, right panels) a juxtacortical T2-hyperintense lesion (upper right panel, lower arrowhead) as well as a new periventricular lesion (lower right panel, right arrowhead) in addition to multiple subcortical lesions (other arrowhead), some with radial orientation (lower right panel, left two arrowheads).

In the fourth group (Cases 9–10), atactic gait was explained by posterior column dysfunction resulting from cervical myelitis. Case 9 had multiple juxtacortical, periventricular, as well as spinal (**Figure 3A**); Case 10 had extensive periventricular and infratentorial white matter lesions (WML) (**Figure 3B**) suggestive for demyelination. Together with corresponding clinical signs and positive oligoclonal IgG in CSF, the diagnosis of MS was made based on current diagnostic criteria (20). Case 10 also exhibited a positive MRZ (measles, rubella, varicella zoster) reaction with elevated CSF/serum antibody indices for measles and varicella zoster typical for MS (25). Of note, Case 6, which had only shown unspecific pericallosal and subcortical WML, also developed progressive periventricular, juxtacortical, and subcortical T2-weighted hyperintense MRI lesions during a follow-up period of five years and was subsequently diagnosed with MS (**Figure 3C**).

**Figure 4:**
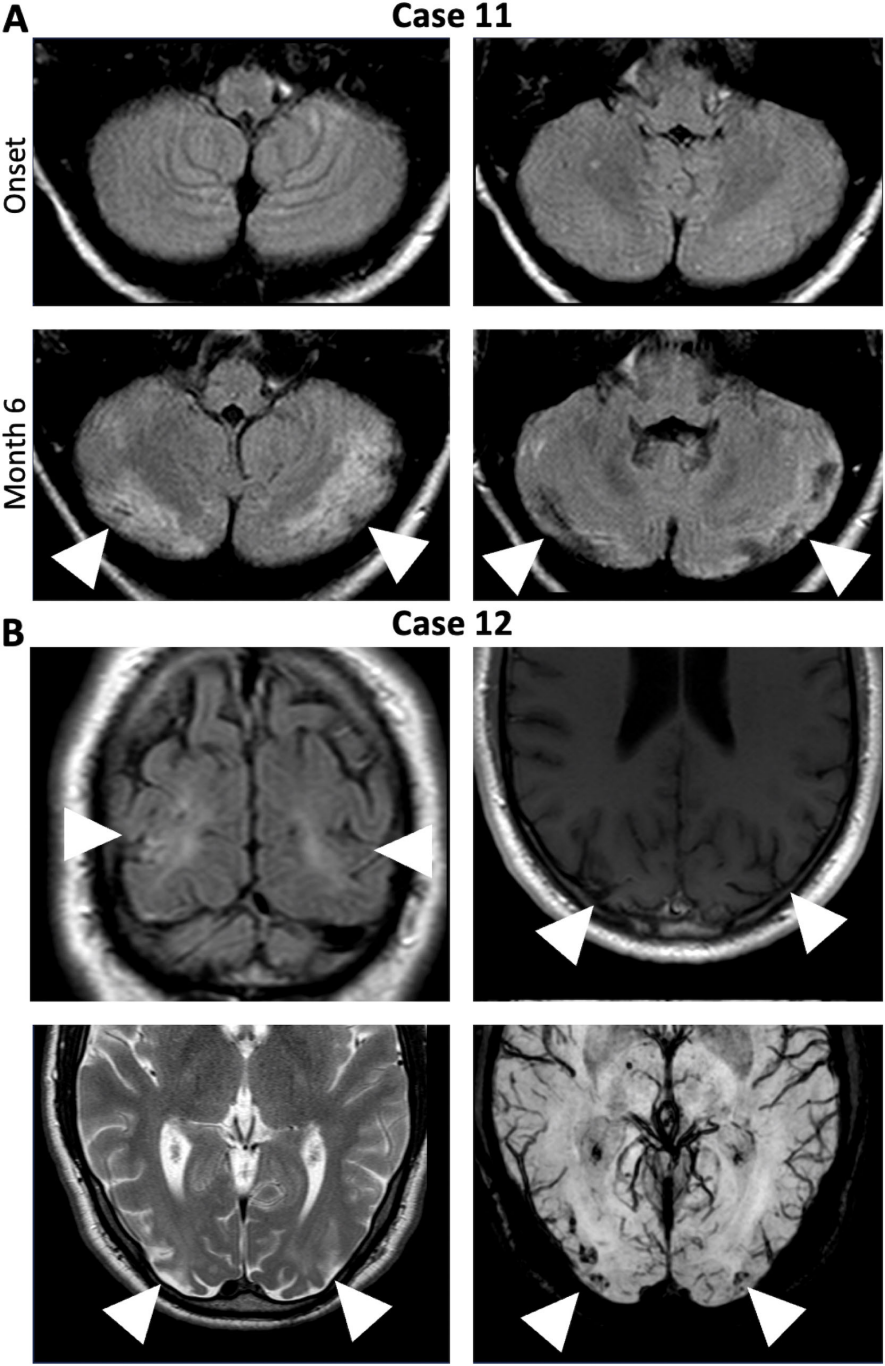
Symptomatic non-cerebellar and cerebellar ataxia due to ischemic hypoxic lesion acquired during circulatory failures in severe NMDAR-E. **(A)** Cerebral FLAIR MRI of Case 11 at onset did not show any cerebellar abnormalities (upper panels). After resuscitated 6 months after disease onset (lower panel, Month 6), repeat cerebral MRI showed bilateral subacute cerebellar infarctions (lower panels, arrowheads). **(B)** Bilateral hemodynamic ischemic lesions in the parietooccipital region (arrowheads) with hemorrhagic transformation (left upper panel: coronal FLAIR, right upper panel: transverse T1-weighted image, left lower panel: transversal T2-weighted image, right lower panel: Transverse susceptibility-weighted images (white arrows) in Case 12 after cardiopulmonary arrest and successful cardiopulmonary resuscitation.

In the fifth group (Cases 11/12), the ataxia, one cerebellar, the other non-cerebellar, resulted from cerebral hypoxic ischemia acquired due to circulatory failure and resuscitation during ICU treatment of severe NMDAR-E (**Figure 4**).

As MOG, AQP-4, KLHL11, and GluK2 antibodies have been identified in NMDAR-E patients and have been associated with neurological symptoms including ataxia (14, 17, 18), we tested available sera for all four antibodies. Serum was tested negative for MOG, AQP-4, KLHL11, and GluK2 antibodies in five of eight patients with cerebellar ataxia (Cases 3–5, 7, and 8). Among these were two of four patients with bilateral cerebellar ataxia without additional CNS findings (Cases 3/4), among those the patient with persistent ataxia, one of the two patients with bilateral cerebellar ataxia with focal CNS findings (Case 5), and both patients with hemiataxia (Cases 7/8, **Table 1**). The three patients meeting the diagnostic criteria for both NMDARE and MS (Cases 6, 9, and 10) were tested negative for both MOG and AQP-4 antibodies (**Table 1**).

In summary, in 4/12 (50%) NMDAR-E patients, ataxia was definitely of a different pathophysiology than the NMDAR-E. In two each, ataxia either was sensory resulting from myelitis in the context of MS or ataxia was explained by hypoxic brain damage as a treatment complication. In two additional patients, our data suggest an etiology distinct from NMDARE. Ataxia was either probably either explained by contralateral status epilepticus and crossed cerebellar diaschisis or, in retrospect, might have been a first manifestation of MS.

In 6/62 patients with cerebellar ataxia (10%), no definite or probable etiology than NMDAR-E itself was identified. These six patients with cerebellar ataxia not explained by other factors than NMDAR-E did not differ from the other 56 patients regarding age [cerebellar ataxia: median 26 years (IQR 18–41 years), other: median years 25 (21–39 years); *p* = 0.7580], sex distribution [female/male: cerebellar ataxia: 6/0 (100%/0%), other: 43/13 (77%/23%); *p* = 0.3280], or outcome [mRS ≤2: cerebellar ataxia: 5/6 (88%) vs. 49/56 (91%), *p* = 0.5799]. When dichotomized into adult and juvenile/childhood NMDAR-E, cerebellar ataxia was not more frequent in children than in adults [age ≥18 years: 5/56 (9%), age <18 years: 1/6 (17%), *p* = 0.4710].

## 4 Discussion

We intended to identify cases with clear NMDAR-E and ataxia in a cohort of predominantly adult NMDAR-E patients and investigated the immunological comorbidities and outcome of patients with ataxia associated with NMDAR-E over a 12-month period. Ataxia during acute NMDAR-E was documented in 12 cases. Cerebellar ataxia without any alternative pathology was identified in 6/12 cases. In the other six patients, definite or probably other pathophysiologies could be demonstrated. In two of these cases, we found sensory ataxia associated with posterior column dysfunction. Although apparently asymptomatic prior to their NMDAR-E diagnosis, both patients met diagnostic criteria for MS in close temporal relation to the NMDAR-E episode. In one additional case, cerebellar ataxia and concurrent focal neurological deficits fulfilled the diagnostic criteria for MS upon long-term follow-up. Thus, in retrospect, the features not typical for NMDAR including ataxia might have been the first manifestation of MS. In two other patients, the ataxia developed due to cerebral hypoxic ischemia, which was only indirectly related to NMDAR-E.

In contrast, among adult NMDARE patients in our total cohort, 10% had symptoms of cerebellar ataxia without another pathology but NMDAR-E explaining the cerebellar dysfunction. A high frequency of ataxia in childhood (15%–20%) has been previously reported, while it is considered rare in adult NMDAR-E patients (< 3%) (6). Our results suggest that cerebellar ataxias may be more common in adult NMDAR-E patients than expected. In more than half of our patients with NMDARE-associated ataxia, we were able to exclude the presence of MOG, AQP-4, KLHL11, and GluK2 antibodies, all of which have previously been associated with both NMDAR-E and CNS neurological deficits including ataxia (14–18). As NMDAR are expressed in the human cerebellum (8) and their inhibition causes cerebellar ataxia (10, 11), transient bilateral cerebellar ataxia in the context of NMDAR-E may result from anti-NMDAR IgG-mediated NMDAR downregulation.

Surprisingly, three of 12 NMDAR-E patients (25%) with documented ataxia developed MS. Demyelinating diseases associated with NMDAR-E have been reported previously (15), but were found to represent concurrent NMOSD or MOGAD. In all our three cases, both AQP-4-positive NMOSD and MOGAD could be excluded. One case with NMDAR-E/MS overlap (Case 9) had documented a positive MRZ reaction, but also a case without MS (Case 8). The MRZ reaction is thought to be the most specific laboratory marker for MS (25). MRZ positivity in NMDAR-E in a single case was first reported by us in 2015 (26). Subsequently, we could demonstrate that the MRZ reaction is positive in one-fifth to one-third of patients with NMDAR-E (27). In addition, isolated cases with both MS and NMDAR-E have been reported (28–32). In synopsis, these results strongly suggest that NMDAR-E is not only the second most common neurological disease in which a positive MRZ reaction occurs but also that co-occurrence of both NMDAR-E and MS may be far more common than expected. This may indicate immunological commonalities between both diseases that need to be investigated.

In some of our other patients with NMDAR-E and ataxia, additional neurological symptoms suggestive of brainstem and optic nerve dysfunction were present. In the absence of demyelinating lesions in these patients and in the absence of MOG, AQP-4, KLHL11, as well as GluK2 antibodies in those tested, the pathophysiological basis of these symptoms remains elusive. However, neurological deficits like hemiparesis have been reported rather frequently in childhood NMDAR-E (6) and thus might represent a less common and underappreciated NMDAR-E symptom in adults alike.

In one of our patients, cerebellar MRI abnormalities were highly suggestive for cerebellar diaschisis due to contralateral status epilepticus (24). Usually, crossed cerebellar diaschisis remains asymptomatic. However, single cases with hemiataxia, as in our case, have been reported (33). In addition to this case, we identified two other patients with ataxia caused by cerebral hypoxic infarction and two more cases with sensory ataxia due to myelitis. Consequently, although ataxia might be a common symptom of adult NMDAR-E, this illustrates that alternative additional causes must be excluded.

Finally, our study confirms that NMDAR-E of the Iizuka-type with persistent cerebellar ataxia and initially progressive cerebellar atrophy following severe NMDAR-E (13) is rare. With exception of our index case, which initiated this project (12), no other case with this peculiar phenotype was discovered. Cerebellar ataxia was transient and not associated with a poor functional outcome in all patients but this case. Thus, in general, cerebellar ataxia does not indicate a poor prognosis in NMDAR-E.

The limitations of our study are its retrospective nature and the limited size of our cohort. This was partially due to our decision to only include patients with at least one year of follow-up, as one of the primary goals was to identify additional patients with persisting cerebellar ataxia leading to a persistently poor functional status. However, our study may be a good starting point to explore the frequency of different phenotypes and origins of ataxia in larger cohorts of NMDAR-E patients. In addition, our unexpected finding that MS might co-occur more often with NMDAR-E than expected requires further investigation.

In summary, our study indicates that cerebellar ataxia occurs more often in adult NMDAR-E than expected previously. Cerebellar ataxia with and without additional neurological symptoms is commonly neither explained by concomitant AQP-4 antibody-positive NMOSD or MOGAD nor KLHL11 or GluK2 antibodies. Thus, it might be directly induced by anti-NMDAR antibodies. We demonstrate that it is mandatory to exclude alternative diagnoses as the origin of ataxia in NMDAR-E. These include complications of intensive care treatment, including resuscitation in severe NMDAR-E. In addition, ataxia and associated focal neurological impairments, including them, might represent the first manifestation of comorbid MS.

## Data Availability

The original contributions presented in the study are included in the article/**Supplementary Material**. Further inquiries can be directed to the corresponding author.

## Members of the GENERATE consortium

Achim Berthele, München TU Neurologie. Aiden Haghikia, Magdeburg UK Neurologie. Aleksandra Juranek, Osnabrück Neurologie. Alexander Emmer, Halle, UK Neurologie. Alexander Finke, Lüneburg Neurologie. Alexandra Philipsen, Bonn, UK Psychotherapie. Amelie Bohn, Aachen UK RTHW Neurologie. Andeas Linsa, Hoyerswerda, Neurologie. Andeas Steinbrecher, Erfurt, Neurologie. Andre Dik, Münster UK Neurologie/Schlafmedizin. Andrea Kraft, Halle Martha-Maria Neurologie. Andreas Binder, Saarbrücken, Neurologie. Andreas van Baalen, Kiel UKSH, Pädiatrie. Anja Tietz, Kiel, UKSH Neuroimmunolo./klein.Chem. Anna Gorsler, Beelitz-Heilstätten, Neuroreha. Anne-Katrin Pröbstel, Basel UK, Neurologie. Annette Baumgartner, Basel UK, Neurologie. Annika Kather, Dresden UK Neurologie. Antonia Harms, Bonn Epileptoloige. Armin Grau, Ludwighafen, Neurologie. Astrid Blaschek, München Haunersches Pädiatrie. Bettina Balint, Zürich, UK Neurologie. Birgit Berger, Rostock, Neurologie. Brigitte Wildemann, Heidelberg, Neurologie. Britta Greshake, Ulm UK Neurologie. Carlos Martinez Quesada, Essen, Neurologie. Carolin Baade-Büttner, Jena UK, Neurologie. Carsten Finke, Berlin Charité, Neurologie/Neurocure. Catharina Groß, Münster UK Neurologie/Schlafmedizin. Christian Bien, Bielefeld Mara, Epilepsiezentrum. Christian Geis, Jena UK, Neurologie. Christian Hofmann, Ulm UK Neurologie. Christian Urbanek, Ludwigshafen, Neurologie. Christine Strippel, Münster UK Neurologie/Schlafmedizin. Christoph Kellinghaus, Osnabrück Neurologie. Christoph Lehrich, Münster UK Neurologie/Schlafmedizin. Christos Krogias, Bochum, St. Josef-Hospital, Neurologie. Chung Ha-Yeun, Jena UK, Neurologie. Claudia Sommer, Würzburg Neurologie. Constanze Kerin (geb. Mönig), Münster UK Neurologie/Schlafmedizin. Corinna Bien, Bad Salzuflen, MVZ Labor Krone. Corinna Trebst, Hannover, MHH Neurologie. Daniel Bittner, Nordhausen, Neurologie. Daniel Rapp, Ulm UK Neurologie. Daniela Esser, Kiel UKSH, Neuroimm./klein. Chemie. Dietrich Sturm. Dirk Fitzner, Göttingen, Neurologie. Dominica Ratuszny, Hannover, MHH Neurologie. Dominique Endres, Freiburg Psychatrie. Duygu Pul, Düsseldorf UK Neurologie. Ellen Knierim, ohne Zentrum (ehem. Berlin, Charite; Pädiatrie). Fabian Möller, Hamburg Asklepios Barmbek, Neurologie. Fatme Seval Ismail, Bochum, KnappschaftsKH, Neurologie. Felix Rosenow, Frankfurt, Epilepsiezentrum & Neurologie. Felix Fischbach, Hamburg Eppendorf, Neurologie. Felix von Poderwils, Greifswald, Neurologie. Florian Then Bergh, Leipzig Neurologie. Frank Hoffmann, Halle Martha-Maria Neurologie. Frank Leypoldt, Kiel, UKSH Neuroimmunolo./klein.Chem. Frank Seifert, Erlangen, Neurologie. Franz Blaes, Gummersbach KKH Neurologie. Franziska Thaler, München LMU Neuroimmunologie/Neurologie. Friedrich Ebinger, Paderborn Neurologie. George Trendelenburg, Mühlhausen, Neurologie. Gerd Meyer zu Hörste, Münster UK Neurologie/Schlafmedizin. Gernot Reimann, Dortmund, Klinikum, Neurologie. Gesa Schreyer, Kiel UKSH, Neuroimm./klein. Chemie. Gregor Kuhlenbäumer, Kiel UKSH, Neuroimm./klein. Chemie. Günter Seidel, Hamburg Nord, Neurologie. Hagen Huttner, Giessen, Neurologie. Harald Prüß, Berlin Charité, Neurologie/Neurocure. Hauke Schneider, Augsburg UK, Neurologie. Hayrettin Tumani, Ulm UK Neurologie. Henning Stolze, Flensburg, Neurologie. Henrik Rohner, Bonn, UK Psychotherapie. Ilya Ayzenberg, Bochum, St. Josef-Hospital, Neurologie. Ina Reichen, Zürich, UK Neurologie. Ina Schröder, Kiel UKSH, Neuroimm./klein. Chemie. Ina-Isabelle Schmütz, Kiel UKSH, Neuroimm./klein. Chemie. Jan Lewerenz, Ulm UK Neurologie. Jan Lünemann, Münster UK Neurologie/Schlafmedizin. Jan Wagner, Ulm UK Neurologie. Jaqueline Klausewitz, Bochum, St. Joseph, Neurologie. Jens Harmel, Kempen Hospital Heiligen Geist, Neurologie. Jens Schaumberg, Lübeck Sana Kliniken, Neurologie. Jens Schmidt, Göttingen, Neurologie. Joachim Havla, München LMU Neuroimmunologie/Neurologie. Johanna Maria Helena Rau, Münster UK Neurologie/Schlafmedizin. Johannes Piepgras, Mainz, UK Neurologie. Jonathan Wickel, Jena UK, Neurologie. Josef Priller, Berlin Charité, Pschologie/Psychatrie. Jost Obrocki, Elmshorn, Psychatrie. Judith Wagner, Gelsenkirchen, Neurologie. Julia Maren Decker, Rüdersdorf, Neurologie. Julian Dominik, LMU München/Großraden. Juliane Spiegler, Würzburg Pädiatrie. Jürgen Hartmut Faiss, Teupitz Neurologie. Justina Dargvainiene, Kiel UKSH, Neuroimm./klein. Chemie. Kai Siebenbrodt, Frankfurt, Epilepsiezentrum & Neurologie. Karin Storm van`s Gravesande, Freiburg, Pädiatrie. Karsten Witt, Oldenburg, Neurologie. Kartharina Wurdack, Berlin Charité, Neurologie/Neurocure. Katharina Eisenhut, München LMU Neuroimmunologie/Neurologie. Kathrin Doppler, Würzburg Neurologie. Katrin Thies, Klinikum Kassel, Neurologie. Kerstin Hellwig, Bochum, St. Josef Hospital, Neurologie. Kevin Rostasy, Datteln, Neuropädiatrie. Kim Kristin Falk, Kiel UKSH, Neuroimm./klein. Chemie. Klarissa Hanja Stürner, Kiel, UKSH Neuroimmunolo./klein. Chem. Klaus-Peter Wandinger, Lübeck UKSH, Neuroimmunolog./klein. Chem.. Kristin Stefanie Melzer, Münster UK Neurologie/Schlafmedizin. Kurt-Wolfram Sühs, Hannover, MHH Neurologie. Lara Zieger, Marburg UK Neurologie. Laura Ehrhardt, Mainz, UK Pädiatrie. Lena Edelhoff, Hamburg Marienkrankhaus, Neurologie. Lena Kristina Pfeffer, Hamburg Eppendorf, Neurologie. Loana Penner, Ulm UK Neurologie. Luise Appeltshauser, Würzburg Neurologie. Makbule Senel, Ulm UK Neurologie. Manuel Friese, Hamburg Eppendorf Neurologie. Marc Nikolaus, Berlin; Charité Pädiatrie. Marcel Gebhard, Halle, Martha-Maria, Neurologie. Marco Gallus, Münster UK Neurologie/Schlafmedizin. Mareike Jansen, Kiel, UKSH Neuroimmunolo./klein.Chem. Mareike Schimmel, Augsburg UK, Neuropädiatrie. Marie Madlener, Köln UK Neurologie. Marie-Luise Mono, Zürich Stadtspital Neurologie. Marina Entscheva, Lübben, Neurologie. Marina Flotats-Bastardas, Homburg/Saar Pädiatrie. Marius Ringelstein, Düsseldorf UK Neurologie. Markus Krämer, Essen Alfried-Krupp KH Neurologie. Markus Rauchenzauner, Vogtareuth, Schön Klinik. Martin Berghoff, Giessen, Neurologie. Martin Häusler, Aachen UK RTHW , Neuropädiatrie. Martin Lesser, Dresden UK Neurologie. Martina Jansen, Kiel UKSH, Neuroimm./klein. Chemie. Mathias Fousse, Homburg/Saar, Neurologie. Matthias von Mering, Bremen Klinikum Nord, Neurologie. Max Kaufmann, Hamburg Eppendorf, Neurologie. Max Vogtmann, Halle, UK Neurologie. Methap Türedi, Ulm UK Neurologie. Michael Adelmann, Weilmünster Neurologie. Michael Karenfort, Düsseldorf UK, Pädiatrie. Michael Malter, Erkelenz, Neurologie. Mona Dreesmann, Potsdam, Pädiatrie. Monika Meister, Ulm, Bundeswehrkrankenhaus. Mosche Pompsch, Essen, Alfried-Krupp Krankenhaus, Neurologie. Muriel Stoppe, Leipzig Neurologie. Nele Retzlaff, Rostock, Neurologie. Nico Melzer, Düsseldorf UK Neurologie. Niels Hansen, Göttingen, Psychatrie. Niels Margraf, UKSH Kiel, Neurologie. Niklas Vogel, Düsseldorf UK Neurologie. Njiku Melchior Wellmer, Ulm UK Neurologie. Olga Simova, Hamburg Alsterdorf, Epilepsie-Zentrum. Oliver Bähr, Aschaffenburg, Neurologie. Oliver Grauer, Münster UK Neurologie/Schlafmedizin. Oliver Stammel, Hamburg Barnbek Neurologie. Patrick Schramm, Giessen, Neurologie. Paul Friedemann, Berlin Charité, Neurologie/Neurocure. Pawel Tacik, Bonn, Neurodegeneration/Gerondopsych. Peter Huppke, Jena UK Pädiatrie. Peter Körtvélyessy, Berlin Charité, Neurologie/Neurocure. Philip Hillebrand, Bonn UK, Pädiatrie. Raphael Reinecke, Frankfurt, Epilepsiezentrum & Neurologie. Regina Trollmann, Erlangen, Pädiatrie. Robert Berger, Hamburg Altona, Neurologie. Robert Handreka, Cottbus, Carl-Thime Klinikum, Neurologie. Robert Rehmann, Dortmund, Klinikum, Neurologie. Robert Weissert, Regensburg, UK Neurologie. Rolf Kern, Worms, Neurologie. Ruth Kerkhoff, Mönchenglatbach, Maria-Hilf. Ruth Schilling, Leipzig, Neurologie. Sarah Bernsen, Bonn, Neurodegeneration/Gerondopsych. Sascha Berning, Osnabrück Neurologie. Saskia Jania Räuber, Münster UK Neurologie/Schlafmedizin. Sebastian Baatz, Altenburg Neurologie. Sebastian Bauer, Frankfurt, Epilepsiezentrum & Neurologie. Sergio Castro-Gomez, Bonn, Neurodegeneration/Gerondopsych. Sigrid Mues, Bochum, KnappschaftsKH, Neurologie. Simon Schuster, Kiel UKSH, Neuroimm./klein. Chemie. Simone Tauber, Aachen UK RTHW Neurologie. Sonka Benesch, Aschaffenburg, Neurologie. Stefan Bittner, Mainz, UK Neurologie. Stefanie Becker, Ulm Neurologie. Steffen Pfeuffer, Giessen, Neurologie. Steffen Syrbe, Heidelberg, Pädiatrie. Stephan Rüegg, Basel UK, Neurologie. Stephan Schreiber, Henningsdorf, Oberhavel Kliniken. Stjepana Kovac, Münster UK Neurologie/Schlafmedizin. Susanne Knake, Marburg UK Neurologie. Sven Ehrlich, Ohne Zentrum (ehem. Wermsdorf). Sven Meuth, Düsseldorf UK Neurologie. Tanja Kümpfel, München LMU Neuroimmunologie/Neurologie. Thanos Tsaktanis, Erlangen, Neurologie. Theodor Rüber, Bonn Epileptoloige. Thomas Grüter, Bochum, St. Joseph, Neurologie. Thomas Pfefferkorn, Ingolstadt, Neurologie. Thomas Seifert-Held, Graz Neurologie. Thorleif Etgen, Traunstein, Neurologie. Til Menge, Düsseldorf, LVR, Neurologie. Timo Deba, Münster, Pädiatrie. Tobias Freilinger, Passau Neurologie. Ulrich Hofstadt-van Oy, Dortmund, Westphalen, Neurologie. Valentin Held, Mannheim UK Neurologie. Verena Kraus, München TU Pädiatrie. Walid Fazeli, Bonn UK, Pädiatrie. Wolfgang Heide, Celle Allg. KH, Neurologie. Yannic Saathoff. Yetzenia Dubraska Haro Alizo, Ingolstadt, Neurologie.
