# Supplementary material for: Frequency, characteristics, and immunological accompaniments of ataxia in anti-NMDAR antibody-associated encephalitis
Source: Front Immunol. 2024 Dec 13;15:1500904. doi: 10.3389/fimmu.2024.1500904 (PMC11681429; doi:10.3389/fimmu.2024.1500904)
Supplement: Supplementary file 1 [file DataSheet1.docx]

**Appendix**

1. **Bilateral Ataxia without additional symptoms**
   1. **Bilateral cerebellar ataxia as first manifestation of NMDAR-E**

**Case 1: Childhood NMDAR-E with cerebellar ataxia as initial symptom**

A 7-year-old developmentally normal girl was admitted with a two-week history of gait instability with subsequent falls. Four days later, she had become increasingly restless and agitated with loss of appetite and sleeping difficulties. At night she had episodes of enuresis and compulsive behavior (e.g., correcting the bedcover in a particular direction). On admission, the patient had developed severe apathy with episodes of irresponsiveness to tactile and acoustic stimulation for several minutes interrupted by intermittent episodes of agitation and aggressive behavior. Neurological examination revealed mild dysarthria, dysmetria in the knee-to-heel maneuver, dysdiadochokinesia and episodes of choreoathetoid movements in both upper limbs. Gait was unstable with internal rotation of the feet. Initially, cerebral MRI and EEG were unremarkable.

As the disease progressed, the patient exhibited episodes of autoaggressive behavior, screaming attacks and short-term memory impairment. Approximately one month after onset of symptoms, she developed bilateral tonic-clonic seizures, some with focal onset on the left side of the body. The seizures lead to recurrent generalized status epilepticus. Episodes of tachycardia up to 180/min were documented, leading to the diagnosis of Wolff-Parkinson-White (WPW) syndrome. Signs of central hyperthermia were also observed. Follow-up cerebral MRI showed T2-hyperintensity and edema in the right hippocampal region. Routine CSF examination was unremarkable with a normal cell count (1 leukocyte/µl), negative oligoclonal bands and an intact blood-CSF-barrier. Anti-NMDAR IgG antibodies were positive in both CSF and serum. Abdominal ultrasound and MRI showed no evidence of ovarian teratoma. The patient received two courses of high-dose methylprednisolone (HDMP, 1g/3 days) followed by oral prednisolone taper. In addition, anti-seizure medication (valproic acid) was administered with benzodiazepines on demand. In response to corticosteroid treatment, seizures remitted and behavior improved. She was referred for neurological rehabilitation. Despite the initial response to corticosteroids, short-term memory impairment persisted as did compulsive behavior and nocturnal agitation. She was treated with mycophenolate mofetil (500 mg x 2 per day) for one year. At follow-up, three years after disease onset, almost complete recovery was observed with mild short-term memory impairment.

**1.2 Bilateral ataxia in adult NMDAR-E**

**Case 2: Delayed detection of NMDAR-E IgG in cerebrospinal fluid in a patient with bilateral cerebellar ataxia**

This 46-year-old female initially presented with a three-month history of assumed postnatal depression after birth of her eighth child. While she had never experienced perinatal psychiatric or neurologic deficits before, she had now developed apathy, lack of energy, sleep disturbances and lack of appetite. After a presyncope, she was admitted to hospital. Cardiovascular screening was unremarkable, but the patient developed severe dysarthria and gait disturbance.

Neurological examination revealed temporal disorientation, lack of motivation, difficulties to concentrate, gaze-evoked nystagmus, gait ataxia with broad-based, small-step gait pattern and limb ataxia of the upper extremities. Reflexes were normal in the upper and lower extremities.

Cerebral and spinal MRI were normal. Sensory evoked potentials were normal, thereby excluding relevant posterior column disorder. Two subsequent CSF analyses showed pleocytosis with 41 and 63 leukocytes/µl, elevated protein (1132 mg/l) and lactate (4.2 mmol/l). Oligoclonal bands were positive. Anti-NMDAR-IgG was negative in two subsequent samples from serum and CSF. Consequently, anti-infective therapy was initiated, but discontinued after receiving negative results for bacterial and viral PCRs.

Cerebral PET CT revealed temporomesial hypermetabolism suggestive but not typical for limbic encephalitis. Whole-body PET CT was not entirely conclusive because of movement artefacts but did not reveal focal hypermetabolism suggestive for a tumour. EEG revealed diffuse slowing.

Despite anti-infective therapy, the patient experienced clinical worsening with increased apathy, aphasia, bizarre behaviour and multiple falls due to severe ataxia. Therefore, HDMP (500 mg over 5 days) followed by oral tapering was started. In the following days, profound clinical improvement was noted. CSF pleocytosis dropped to 22 leukocytes/µl and CSF protein to 654 mg/L, lactate dropped to 3.8 mmol/l. Astonishingly, CSF now tested positive for anti-NMDAR IgG (titre 1:10). Treatment was complemented by a three-day course of IVIG (30 g per day). The patient became able to communicate and walk independently. However, she attempted suicide and reported optic hallucinations. Antipsychotic and antidepressant therapy was adjusted.

In the following weeks and during slow tapering of oral prednisolone, neurological and psychiatric symptoms resolved completely. CSF cell count was 8/µl and anti-NMDAR IgG negative, lactate however was again increased to 3.8 mmol/l. EEG was normal.

After discontinuation of prednisolone, another episode of severe agitation occurred and after few weeks, ataxia reoccurred. CSF analysis revealed a slightly increased pleocytosis of 15 leukocytes/µl and anti-NMDAR IgG again was positive (1:10), lactate dropped to 2.6 mmol/l. EEG showed diffuse slowing. We increased oral prednisolone and IVIG repeated for two more cycles four weeks apart (30 g/d over 5 days, respectively). Treatment with azathioprine was started but had to be discontinued due to hepatotoxicity. Oral prednisolone was tapered to 5 mg/d. Cerebral MRI remained normal during the whole course of the disease.

Six months later and one year after first onset of neurological symptoms, the patient was asymptomatic, CSF cell count was normal (3/µl) and anti-NMDAR IgG in CSF negative, lactate remained slightly elevated with 2.6 mmol/l.

**Case 3: Delayed gait ataxia in therapy-refractory NMDAR-E**

The 29-year-old female patient was admitted following a 6-week history of anxiety, agitation, and psychotic symptoms, cognitive deficits, and bilateral epileptic seizures. At the time of admission, NMDAR-E had already been diagnosed due to the typical clinical presentation and presence of NMDAR antibodies both in the CSF (>1:64) and serum (1:200). HDMP, IVIG and an initial dose of 1000 mg rituximab had already been administered with limited improvement. For the symptomatic treatment of ongoing severe psychiatric symptoms, anxiolytic therapy with benzodiazepines (Lorazepam 5 x 1 mg day) had been initiated.

Upon admission, the patient was disoriented, exhibited reduced alertness and moderate psychomotor slowing along with severe difficulties to concentrate. Speech was slightly dysarthric. Furthermore, significant gait unsteadiness was observed during both normal gait testing and tandem walking. The Romberg test result was positive. Muscle tone and reflex levels of the upper and lower extremities were weak, although no decrease in muscle strength was detected. No further signs of posterior column disorder were documented. Brain MRI revealed slight supratentorial brain volume reduction without any further pathological findings. EEG showed signs of reduced alertness with no clearly distinguishable baseline rhythm. Repeated serum testing again showed positive NMDAR antibodies (1:32). Oral corticosteroid therapy was continued, and a second dose of 1000 mg rituximab administered. Quetiapine and risperidone were started to reduce benzodiazepine intake. Upon neurological examination before discharge, the patient's stance and gait were unimpaired. Reflexes were found to be normal. Due to the transient nature of the most possibly cerebellar ataxia, an adverse effect of the benzodiazepine medication was discussed. Serum of this patient in the period of active NMDAR-E was retrospectively tested negative for MOG, AQP-4, KLHL11 as well as GluK2 antibodies.

Upon follow-up examination 6 months later, the patient reported to be seizure-free with substantial improvement in psychiatric symptoms and only mild memory disturbances remaining as the only symptom. Upon examination, no neurological deficits were noted, especially no stance or gait ataxia. Although NMDAR antibodies remained detectable in serum, in the absence of any relevant remaining deficits two years after the initial presentation immunotherapy was discontinued and anticonvulsants were started to be reduced.

**Case 4: Delayed severe persistent cerebellar ataxia associated with cerebellar atrophy developed during severe NMDAR-E**

This patient was described in detail in a previous case report (1). In summary, this 23-year-old female presented with a typical NMDAR-E with psychotic symptoms, which remained refractory to immunosuppressive therapy and after deterioration with severe autonomic dysregulation required prolonged ICU treatment and multidrug analgosedation. Multidrug analgosedation lead to hepatotoxicity and thus was switched to long-term isoflurane sedation. This was associated with imaging signs of cerebral mitochondrial toxicity. Discontinuation of isoflurane exacerbated autonomic dysregulation including blood pressure as a possible cause of posterior reversible encephalopathy syndrome. The patient survived repeated episodes of sepsis leading to critical illness polyneuropathy/myopathy (CIP/CIM). When she finally improved, in addition to preexisting cognitive and behavioral abnormalities and flaccid distal tetraparesis and sensory dysfunction because of CIP/CIM, severe cerebellar ataxia was noted. Serum of this patient in the period of active NMDARE was retrospectively tested negative for MOG, AQP-4, KLHL11 as well as GluK2 antibodies.

While all other symptoms improved, cerebellar ataxia persisted and was associated with delayed severe cerebellar atrophy. Up to five years later, the patient still suffered from severe cerebellar ataxia with cerebellar dysarthria and limb ataxia with moderate improvement upon intense and continuous physiotherapy. Leg ataxia is functionally exacerbated by the mild residual sensory deficits resulting from CIP/CIM. Therefore, she is still wheelchair-bound. However, no relevant cognitive dysfunction persists and the patient, despite for persisting motor disability including fine motor skills, i.e. writing and typing, regained her ability to proceed with her university degree in economics.

**2 Adult NMDAR-E with cerebellar ataxia and other focal neurological deficits**

**2.1 Focal deficits including bilateral cerebellar ataxia preceding NMDAR-E**

**Case 5: Monoocular blurred vision followed by cerebellar ataxia prior to NMDAR-E**

A 39-year-old female patient presented with a 18-month history of blurred vision on the left eye and initially unsystematic dizziness that had rapidly worsened to a staggering vertigo prohibiting the patient to walk freely. Upon neurological examination, memory deficits and an affective lability became apparent. Pursuit eye movements were saccadic. Speech was dysarthric. Finger-to-nose and heel-to-shin tests were both normal, but there was bradydiadochokinesia of the left upper extremity. In addition, trunk ataxia as well as an unsteady broad-based gait were observed.

Cerebral MRI and EEG were normal. CSF cell count was normal, oligoclonal IgG was negative in CSF. Anti-NMDAR IgG was positive in both serum (1:800) and CSF (1:10). FDG-PET CT showed no evidence of metabolically active neoplasia. Gynecological examination excluded ovarian teratomas and other space-occupying lesions. Serum of this patient in the period of active NMDARE was retrospectively tested negative for MOG, AQP-4, KLHL11 as well as GluK2 antibodies.

The patient received 1000 mg HDMP intravenously over 5 days. Already during the infusions, the patient's gait improved, the stance/gait variations were still unsteady but possible. Following HDMP, oral prednisolone (initially 70 mg) was given and slow tapering advised. At a daily dose of 25 mg prednisolone, the patient was re-admitted due a relapse of gait instability and a weak and clumsy right hand. The gait pattern was again unsteady and broad-based with a tendency to fall backwards. The subjectively reported new onset of weakness of the right hand with additional coordination disturbance cannot be confirmed in the examination. The patient again complained of increased affective lability.

HDMP was repeated for another five days, during which the symptoms subsided rapidly. Long-term immunosuppression, first with azathioprine followed by mycophenolate mofetil was started. Both, however, had to be discontinued due to side effects. Thus, the patient was treated monthly courses of intravenous HDMP (1000 mg). After one year under this regimen, the patient again relapsed with increased unsteadiness of gait. Switching to rituximab was followed almost complete remission. Rituximab could be discontinued after about 5 years without any further relapse.

**Case 6: NMDAR-E with early gait ataxia and dysarthria associated with impaired fine motors skills of the left hand and cranial nerve/brainstem dysfunction and later diagnosis of multiple sclerosis**

This 21-year-old female patient was admitted after one week of malaise and reduced performance capability. She then had developed slurred speech, clumsiness of the left and as well as numbness of the palate. Upon neurological examination, she showed dysarthria. In addition, hypesthesia of the palate and a discrete facial palsy on the right side were noted. Fine motor skills of the left hand were impaired. Slight gait ataxia with increased swaying with eyes closed was present. Deep tendon reflexes were normal. Sensory function including pallaesthesia was normal. In the days after admission, cognitive impairment as well as behavioral changes with increased anxiety developed.

Cerebral MRI was normal. CSF analysis showed pleocytosis (53 leukocytes/µl) as well as isolated oligoclonal bands. Anti-NMDAR IgG was detected in CSF, but not in serum. HDMP was not followed by any clinical improvement. Instead, the patient developed epileptic seizures, which subsided upon antiepileptic treatment with levetiracetam. Following immunoadsorption and IVIG (2g/kg bodyweight), the patient’s cognitive performance improved. One month after initial admission, gait had become normal. The patient was subsequently transferred for rehabilitation treatment.

Upon MRI follow-up, additional periventricular, juxtacortical and subcortical T2-weigthes hyperintense lesions with Dawson finger-like orientation developed. MS diagnosis was subsequently established based on 2017 McDonald criteria 5 years after onset of NMDAR-E.

**3 Hemiataxia associated with NMDAR-E**

**3.1 NMDAR-E with early hemiataxia with other focal neurological deficits**

**Case 7: Adult NMDAR-E with initial focal neurological deficits including hemiataxia**

The 21-year-old female was transferred from another hospital after having developed dizziness, psychomotor slowing and double vision following gastrointestinal symptoms. Cerebral MRI had already demonstrated left frontal and temporoparietal areas with cortical diffusion restriction with contrast enhancement. CSF analysis had shown pleocytosis with 9 leukocytes/µl. An empirical treatment comprising corticosteroids and acyclovir had already been started.

Neurological examination upon admission revealed psychomotor slowing. The patient was disoriented. Upon cognitive screening using MOCA, she scored 12 out of 30 points (norm >26 points). Gaze-evoked nystagmus was present to the right and the left. There was pronator drift of the right arm. Left-sided hemiataxia more pronounced on the leg was present. The finger-to-nose and heel-to-shin tests on the left side both showed dysmetria, the latter was additionally atactic. Babinski sign was negative. No sensory impairment was noted.

Repeat CSF examination showed normal leukocyte count. Two IgG bands isolated in CSF were detected. Anti-NMDAR IgG was negative in serum but positive in CSF (IgG 1:5). While EEG initially showed bifrontal delta slowing, later a pattern suggestive for extreme delta brush became apparent. Gynecological evaluation revealed ovarian teratoma, which was removed by partial ovariectomy. Serum of this patient in the period of active NMDARE was retrospectively tested negative for MOG, AQP-4, KLHL11 as well as GluK2 antibodies.

The patients received IVIG (2 g/kg body weight over 5 days) followed by 375 mg/m² rituximab weekly for four weeks. As fluctuations of attention and orientation were assumed of epileptic origin, levetiracetam was given.

Repeat cerebral MRI showed marked regression of the diffusion restrictions. Clinically, no focal neurological deficits were detectable at discharge, while cognitive impairment was still present (MOCA test 20/30 points).

Upon the follow-up, the patient reported incomplete retrograde amnesia for the acute phase of the disease, persistent word-finding difficulties as well as impaired ability to concentrate. No focal neurological deficits were detected. Anti-NMDAR IgG in CSF now was negative.

No further immunosuppressive treatment was initiated. Eighteen months after onset and following neurological rehabilitation, the patient was able to resume her work in media informatics full-time.

**3.2 NMDAR-E with delayed hemiataxia**

**Case 8: Adult NMDAR-E with extensive cerebellar lesion as a possible correlate of cerebellar diaschisis for right frontal status epilepticus and delayed left-sided hemiataxia**

Two months prior to admission, this 18-year-old female had presented to an external hospital with a first-ever epileptic seizure as well as short episodes of anomia. Diagnostic work-up including cranial MRI, CSF examination, EEG, and testing of anti-NMDAR IgG had revealed no abnormalities. Then, she had been admitted to the department of psychiatry due to personality changes as well as suicidal thoughts. CSF examination had shown 43 leukocytes/µl. Cerebral MRI had revealed extensive right frontal and left cerebellar T2-hyperintensities, predominantly cortical but also extending into the underlying white matter (Figure 2). HDMP (1g/ 3 days) treatment had already been started before the patient was transferred to the respective GENERATE site.

Upon admission she presented with confusion, memory deficits, decreased motivation, as well as suicidal thoughts. Neurologic examination revealed cerebellar oculomotor dysfunction including gaze-evoked nystagmus bilaterally, upbeat nystagmus as well as hypometric saccades. Dysmetria of the left arm was noted. Repeat CSF examination showed that leukocyte count had almost normalized (5/µl). CSF-specific OCB were positive. A polyspecific intrathecal immune reactivation with increased CSF/serum indices for rubella AI (1.8) and HSV (1.6) indicating an intrathecal synthesis was found. Anti-NMDAR IgG was positive in both serum and CSF (both 1:32). Aquaporin-4-Abs were negative. Serum of this patient in the period of active NMDARE retrospectively also tested negative for MOG, KLHL11 as well as GLuK2 antibodies.

EEG revealed right frontal status epilepticus. Therefore, the left cerebellar lesion was interpreted as correlate of cerebellar diaschisis. The patient was transferred to the ICU. Phenytoin was added to the preexisting antiepileptic regimen consisting of valproate and levetiracetam. Repeat HDMP (1g/ 3 days) followed by oral corticosteroids transiently improved agitation, confusion, and memory function. No tumor was identified. However, she then developed catatonia associated with increased anti-NMDAR IgG titers (serum 1:320, CSF 1:100). She was treated with seven cycles of plasma exchange followed by treatment with rituximab (2x1g with an interval of three weeks). A marked clinical improvement ensued. Thus, rituximab was continued (3 times 500 mg every 6 months). Within 1.5 years after onset, anti-NMDAR IgG became negative in serum, within two years in CSF. Two years after onset, OCB became negative. Repeat cerebral MRI showed complete regression of right-sided frontal and left-sided cerebellar lesions with only residual unspecific white matter lesions frontally. With time clinical symptoms remitted completely. Although she had to change her profession, the patient became able working full-time.

**4 Other causes of ataxia**

**4.1 Sensory ataxia associated with demyelinating lesions sufficient to diagnose multiple sclerosis associated with NMDAR-E**

**Case 9: Sensory ataxia due to first clinical manifestation of multiple sclerosis in close temporal association with adult NMDAR-E**

This 59-year-old male presented with a one-week history of progressive weakness and impaired coordination of all four extremities. Beforehand, he had experienced a bout of chills, nausea, and vomiting. Upon neurological examination, mild tetraparesis, left more than right, more pronounced regarding the legs was noted. Deep tendon jerks were more pronounced on the left. The plantar reflex was extensor on the left. A fine motor skills impairment of the left hand was found. Romberg test was positive with loss of balance upon closing his eyes. Urinary retention developed later.

Cerebral MRI showed numerous white matter lesions suggestive for focal demyelination, located juxtacortically and periventricularly including the corpus callosum. Spinal MRI revealed two acute myelon lesions on the level of cervical vertebrae 3/4 and thoracic vertebra 7. CSF showed 80 leukocytes/µl and isolated oligoclonal bands in the CSF and a slightly increased CSF/serum antibody index (AI) for varicella zoster (VZV, 1.5, upper normal value 1.4). Antineuronal antibodies, including NMDAR antibodies, were neither found in serum nor CSF. Serum of this patient in the period of active NMDARE was retrospectively tested negative for MOG und AQP-4 antibodies. Tibial sensory evoked potentials (SEP) were absent, median SEP were normal, visually evoked potentials were normal. Central tetraparesis and sensory ataxia due to myelitis as first clinical presentation of multiple sclerosis were diagnosed and relapse treatment with HDMP (1g/5 days) was started.

Three days after HDMP, the patient started to report suicidal thoughts. He became increasingly disoriented and developed delusions of poisoning. Then he developed episodes of irresponsiveness associated stereotypical head shaking. Although corticosteroid-induced psychosis was assumed as the most likely diagnosis, repeat lumbar puncture was performed. Now pleocytosis had reached 101 leukocytes/µl, quantitively relevant intrathecal IgG and IgM synthesis (18% and 63%, respectively) had developed. In addition, the VZV AI was increased (again 1.5), as well as the measles AI was now increased to 1.7. Repeat examination of antineuronal antibodies now showed positive Anti-NMDAR IgG (1:100) in CSF only.

Following an additional plasma exchange, tetraparesis, sensory ataxia as well as the psychotic symptoms receded. However, his cognitive abilities remained somewhat impaired.

Two months after onset of first symptoms, anti-NMDAR IgG titers were 1:100 in serum and 1:10 in CSF. Another two months later and associated with additional clinical improvement, NMDAR antibodies were again positive in CSF only.

**Case 10: Sensory ataxia due to first clinical manifestation of multiple sclerosis in close temporal association with adult NMDAR-E**

One year after having developed repeated episodes of vertigo and panic attacks, this 40-year-old female presented with gait instability that rapidly progressed within days leading to falls and finally inability to walk or even stand. In association with the loss of stance, urinary and fecal incontinence but also diplopia and slurred speech developed. Neurological examination revealed psychomotor slowing and cognitive dysfunction. The patient had dysarthrophonia. Motor examination revealed distally pronounced tetraparesis. There was hypaesthesia distally from the level of thoracic vertebra 8 associated with sensory ataxia.

In the past, cerebral MRI showed numerous acute white matter lesions suggestive for focal demyelination, located supra- and infratentorial and including the pons and myelon at level of cervical vertebra 2. The CSF examination revealed a pleocytosis of 19 leukocytes/µl as well as isolated oligoclonal bands in CSF. Retrospectively, both MOG and AQP-4 antibodies were tested negative in serum of this patient. Myelitis as first clinical manifestation of multiple sclerosis was diagnosed and treatment with HDMP (1g/5 days) initiated. As no improvement was noted, plasma exchange was performed. As plasma exchange did not show any effect, cyclophosphamide (750 mg/m² body surface two times) was administered, which finally was associated with clinical improvement. Following rehabilitation, the patient regained her ability to walk.

A few months later, the patient relapsed again presenting with transverse spinal cord syndrome with tetraparesis, sensory impairment as well as with urinary and fecal incontinence. Again, dysarthria as well as psychomotor slowing and cognitive impairment were noted. Repeat HDMP as well as plasma exchange failed to lead to improvement as did rituximab (1000 mg). Repeat examination of antineuronal antibodies showed positive NMDAR antibodies (titer 1:10 in serum, CSF not tested, retrospectively confirmed in post-acute serum two years after first detection using immunohistochemistry on rat brain). A tumor search via PET-CT remained inconspicuous. As the patient failed to improve, bilateral ovariectomy was performed. However, no teratoma could be detected. Following ovariectomy, psychomotor slowing and cognitive dysfunction remitted. However, tetraparesis only moderately improved. Thus, cyclophosphamide (750 mg/m² body surface) was repeated for several cycles. Although a baclofen pump was implanted for persistent paraspastic the following year, the patient remains wheelchair-bound due to spasticity and persistent sensory ataxia.

**4.2. Ataxia due to cerebral hypoxic ischemia acquired during circulatory failure in severe NMDAR-E**

**Case 11: Ataxia due to bilateral cerebellar infarctions during a prolonged ICU stay due to NMDAR-E**

This 20-year-old female patient was admitted with a one-week-history of behavioral changes with pronounced irritability followed by a generalized tonic-clonic epileptic seizure. After admission, she developed psychiatric symptoms with suicidality and became disoriented. Epileptic seizures became more and more frequent. Thus, the patient was transferred to the ICU. Ten days after admission, the patient had to be intubated following loss of consciousness and central hypoventilation. Anti-NMDAR IgG was detected in both serum and CSF. CSF showed pleocytosis with 80 leukocytes/µl. Cerebral MRI was normal. EEG showed intermittent frontotemporal delta dysrhythmias and delta brushes. Upon the exploratory partial ovariectomy, no teratoma was found.

Treatment consisted of HDMP, plasma exchange, rituximab and finally cyclophosphamide. Within 185 days of ICU treatment, the patient only slowly regained consciousness, developed massive hyperkinesias, autonomic disturbances (hypothermia), focal and bilateral tonic-clonic seizures as well as status epilepticus. As complications of ICU treatment, the patient had several episodes of sepsis, developed acute respiratory distress syndrome and had to be resuscitated once.

Repeat cerebral MRI four months after admission showed bilateral subacute cerebellar (hypoxic) infarctions. The clinical correlate thereof was bilateral ataxia with dysmetria in the Bárány test and atactic gait. In the following months and during neurological rehabilitation, ataxia steadily improved. However, the upper limbs still showed dysmetria and gait remained atactic upon follow-up. A full recovery of ataxia of the upper limb was seen 2 years after initial hospital admission. The gait normalized 3 years after onset of NMDAR-E**.**

**Case 12: 33-year-old patient with anti-NMDAR encephalitis and delayed bilateral ataxia following with central arm paresis period of reduced consciousness (KIE-0017)**

A 33-year-old male patient presented with right-hemispheric focal to bilateral tonic-clonic seizures followed by short-term memory deficits, difficulties to concentrate, aphasia and executive dysfunction. Cerebral MRI did not show any abnormalities. Upon CSF analysis, leukocyte count was normal (0 leukocytes/µl) without isolated oligoclonal bands. Anti-NMDAR IgG was positive in both CSF (1:32) and serum (1:320). Serum of this patient in the period of active NMDARE was retrospectively tested negative for MOG and AQP-4 antibodies. The patient received HDMP (1 g for 5 days) and anti-epileptic medication with valproic acid and levetiracetam. The patient developed hallucinations, which were treated with haloperidol. As no clinical improvement ensued in response to corticosteroids, plasma exchange with 5 sessions was performed. Unexpectedly, the patients experienced a cardiopulmonary arrest, probably due to second-degree atrioventricular block (Mobitz type I). Cardiopulmonary resuscitation was successful within 15 minutes. However, during the following ICU treatment, tracheotomy became necessary due to prolonged weaning. A cardiac pacemaker was implanted. Additional five sessions of plasma exchange were performed. A follow-up brain MRI showed bilateral hemodynamic hypoxic ischemic lesions in the occipital and central regions, interpreted as a result of hypotension during cardiopulmonary arrest. Neurological examination revealed mild central paresis of the left arm (muscle strength 4/5) with slightly exaggerated left reflex status. The gait was wide-based. Romberg test was positive with considerable sway. Superficial perception and pallesthesia were normal. The gait instability was suspected to be caused by a hemodynamic stroke. After intensive care and the treatment with HDMP and plasma exchange described above, the seizures stopped and there was a marked improvement in short-term memory, aphasia and executive dysfunction. At 2-year follow-up, the patient reported only mild executive dysfunction and was able to return to work as a bank clerk. Complete recovery was reported at the 4-year follow-up.

1. Jesse S, Wagner J, Gastl R, Steinacker P, Otto M, Kassubek J, et al. On Razor's edge: Managing analgosedation during severe anti-NMDA receptor encephalitis. Neurol Neuroimmunol Neuroinflamm. 2019;6(1):e522.
