# Supplementary material for: Frequency, characteristics, and immunological accompaniments of ataxia in anti-NMDAR antibody-associated encephalitis
Source: Front Immunol. 2024 Dec 13;15:1500904. doi: 10.3389/fimmu.2024.1500904 (PMC11681429; doi:10.3389/fimmu.2024.1500904)
Supplement: Supplementary file 2 [file Table1.docx]

Supplementary Table 1 : Routine testing for antineuronal antibodies upon clinical work-up of the twelve patients with NMDARE and ataxia

| **Pat #** | **Hu** | **Yo** | **Ri** | **Ma/Ta** | **CV2** | **Amph** | **Sox1** | **Zic-4** | **Tr** | **VGCC** | **AMPAR** | **GABABR** | **LGI1** | **CASPR2** | **VGKC** | **DPPX** | **GAD** | **GlyR** |
| --- | --- | --- | --- | --- | --- | --- | --- | --- | --- | --- | --- | --- | --- | --- | --- | --- | --- | --- |
| **1** | n.a. | n.a. | n.a. | n.a. | n.a. | n.a. | n.a. | n.a. | n.a. | n.a. | n.a. | n.a. | n.a. | n.a. | n.a. | n.a. | n.a. | n.a. |
| **2** | neg. | neg. | neg. | neg. | neg. | neg. | n.a. | n.a. | n.a. | n.a. | neg. | n.a. | n.a. | n.a. | n.a. | n.a. | neg. | n.a. |
| **3** | neg. | neg. | neg. | neg. | neg. | neg. | neg. | n.a. | neg. | n.a. | neg. | neg. | neg. | neg. | n.a. | neg. | neg. | n.a. |
| **4** | neg. | neg. | neg. | neg. | neg. | neg. | neg. | neg. | neg. | n.a. | neg. | neg. | neg. | neg. | n.a. | neg. | neg. | n.a. |
| **5** | neg. | neg. | neg. | neg. | n.a. | neg. | neg. | neg. | n.a. | n.a. | neg. | neg. | neg. | neg. | n.a. | n.a. | neg. | n.a. |
| **6** | neg. | neg. | neg. | neg. | neg. | neg. | neg. | n.a. | n.a. | neg. | neg. | neg. | neg. | neg. | n.a. | neg. | n.a. | n.a. |
| **7** | neg. | neg. | neg. | neg. | neg. | neg. | neg. | neg. | neg. | n.a. | neg. | neg. | neg. | neg. | neg. | n.a. | neg. | n.a. |
| **8** | neg. | neg. | neg. | neg. | neg. | neg. | neg. | n.a. | n.a. | n.a. | neg. | neg. | neg. | neg. | n.a. | neg. | neg. | n.a. |
| **9** | neg. | neg. | neg. | neg. | neg. | neg. | neg. | neg. | neg. | n.a. | neg. | neg. | neg. | neg. | n.a. | neg. | neg. | n.a. |
| **10** | neg. | neg. | neg. | neg. | neg. | neg. | neg. | neg. | neg. | n.a. | neg. | neg. | neg. | neg. | n.a. | n.a. | neg. | neg. |
| **11** | neg. | neg. | neg. | neg. | neg. | neg. | n.a. | n.a. | n.a. | n.a. | n.a. | n.a. | n.a. | n.a. | n.a. | n.a. | n.a. | n.a. |
| **12** | neg. | neg. | neg. | neg. | n.a. | neg. | n.a. | n.a. | neg. | n.a. | neg. | neg. | neg. | neg. | n.a. | n.a. | neg. | neg. |

Pat # = patient number, Amph = Amphiphysin, VGCC = voltage-gated calcium channels, VGKC = voltage-gated potassium channels, GlyR = glycine receptor, neg. = negative, n.a. = not analyzed-
